# Case Report: Late Reactivation of Herpes B Virus After a Monkey Bite: A Case of Severe Meningoencephalitis

**DOI:** 10.4269/ajtmh.23-0253

**Published:** 2023-10-16

**Authors:** Ester Ponzetto, Quentin Delhez, Maarten Hoppenbrouwers, Nicolas De Schryver, Nicolas Serck, Thierry Dugernier, Marie-Céline Duray, Benjamin Gressens, Marco Vinetti, Gregorius J. Sips, Jeroen van Kampen, Corine H. GeurtsvanKessel, Sander van Boheemen

**Affiliations:** ^1^ Intensive Care Unit, Clinique Saint-Pierre, Ottignies, Belgium;; ^2^ Department of Viroscience, Erasmus MC, Rotterdam, The Netherlands;; ^3^ Department of Neurology, Clinique Saint-Pierre, Ottignies, Belgium;; ^4^ Public Health Service Rotterdam-Rijnmond, Rotterdam, The Netherlands

## Abstract

*Macacine alphaherpesvirus* 1, also known as herpes B virus (BV), is an alphaherpesvirus endemic to several macaque species, capable of causing zoonotic infections in humans, with high mortality rates. Evidence of reactivation in humans has rarely been reported. Here we depict a case of BV reactivation after 54 years, leading to severe meningoencephalitis. This case supports the use of antiviral prophylaxis in patients surviving a confirmed BV central nervous system infection. We sequenced DNA from BV obtained from the patient’s cerebrospinal fluid. Phylogenetic analysis showed significant divergence in the clustering of this particular BV strain compared with other known BVs. Therefore, additional efforts are needed to obtain a broader sequence landscape from BVs circulating in monkeys.

## INTRODUCTION

*Macacine alphaherpesvirus* 1 (McHV-1), or herpes B virus (BV), is an alphaherpesvirus endemic to certain macaque species. It is the only one of nearly 35 identified nonhuman primate herpesviruses that is known to cause zoonotic infections in humans.^1^ Although it causes only localized cutaneous or mucosal lesions in its natural host, it is neurotropic and neurovirulent in the foreign human host.^1^ BV is a significant occupational hazard for people working with macaques, but it may also be a concern for tourism, zoos, and the growing illegal pet trade.^2^

Human infection occurs after exposure to infected body fluids during the handling of macaque monkeys, which are often used in biomedical research.^2^^,^^3^ If untreated, BV has an extremely high mortality rate (∼80%) in humans.^1^

Human symptomatic infection with BV seems rare, and the existing literature on this type of infection comprises only a few case reports. Since the first case, reported in 1933,^4^ only 50 cases have been identified in the United States, with many being incompletely documented.^2^ The number of case reports peaked in the late 1950s, when the battle against human poliovirus infection led to an unprecedented use of rhesus monkeys (*Macaca mulatta*) in biomedical laboratories.^1^^,^^5^^–^^12^

Being characterized as one of the alphaherpesviruses, BV can, in its natural host, periodically reactivate from the latent state in response to various stressful stimuli, leading to renewed viral shedding.^13^ Yet little is known about the risk of interhuman transmission and human reactivation with BV infection.^13^

To our knowledge, we here report the first case of BV reactivation manifesting as severe meningoencephalitis 54 years after primary infection.

## CASE PRESENTATION

**Figure 1.**
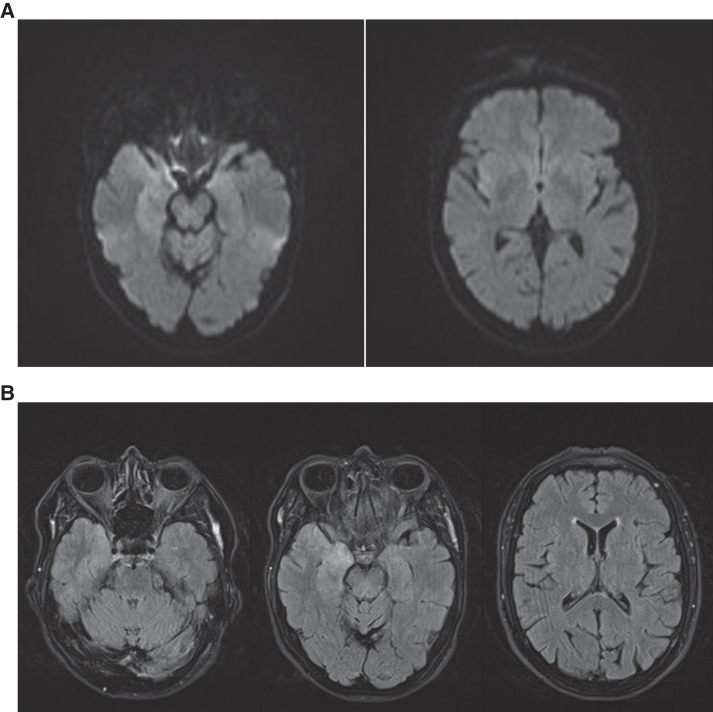
Brain magnetic resonance imaging (MRI) on day 2. (**A**) Brain MRI, DWI: hyperintense signals in the right inferior temporal gyrus and juxta insular. (**B**) Brain MRI, FLAIR: hyperintense signals in the same parts of the DWI sequences.

A 66-year-old woman was admitted to the hospital presenting with fever and drowsiness for 48 hours. She had no history of chronic diseases or medication use; however, recurrent herpes labialis was reported. Upon hospitalization, she remained febrile, with a temperature of up to 39.5°C. She remained conscious and responsive to verbal commands but was apathetic. Neck stiffness was not observed. The initial diagnostic workup did not reveal an etiological agent (Supplemental Table 1). On day 3, a lumbar puncture was performed, which revealed a lymphocytic pleocytosis with mild hyperproteinorrachia. Viral meningoencephalitis was suspected and treatment with 10 mg/kg acyclovir three times a day (TID)^14^ was started while awaiting the diagnostic results (Supplemental Table 1). Brain computed tomography followed by magnetic resonance imaging (MRI) was performed on the same day and showed, on fluid-attenuated inversion recovery (FLAIR) sequence, on diffusion-weighted imaging (DWI) and echo planar sequences a discrete hyperintensity of the right inferior temporal gyrus and juxta insular. These radiological images were suggestive of a temporal inflammatory or infectious encephalitis (Figure 1).^15^ On day 4, the patient was transferred to the intensive care unit as she gradually developed mutism and a dystonic posture with cervical dystonia and flexion of the four limbs without signs of lateralization. Subsequently, we noted an altered level of consciousness as defined by a Glasgow Coma Scale of 7 out of 15. By day 5, she required intubation and mechanical ventilation. Epileptic waves were observed on repeated electroencephalograms, for which a treatment with valproic acid and levetiracetam was initiated. Herpes simplex virus 1 (HSV-1) DNA, herpes simplex virus 2 (HSV-2) DNA, varicella zoster virus (VZV) DNA, and enterovirus RNA remained undetectable by polymerase chain reaction (PCR) on repeated cerebrospinal fluid (CSF) samples. On day 9, repeated brain MRI showed worsening of the lesions on the FLAIR sequence with involvement of the medium temporal cortex, the parietal cortex, and the right thalamus. The increase in hyperintensity on DWI in these regions suggested cytotoxic edema, likely resulting from status epilepticus. On day 10, in response to escalating pleocytosis in CSF, the acyclovir dosage was increased to 15 mg/kg TID (Supplemental Table 1).^16^

**Figure 2.**
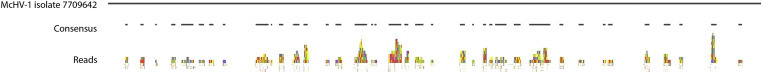
Coverage of the metagenomic sequencing reads across the *Macacine alphaherpesvirus* 1 (McHV-1) isolate 7709642 genome.

**Figure 3.**
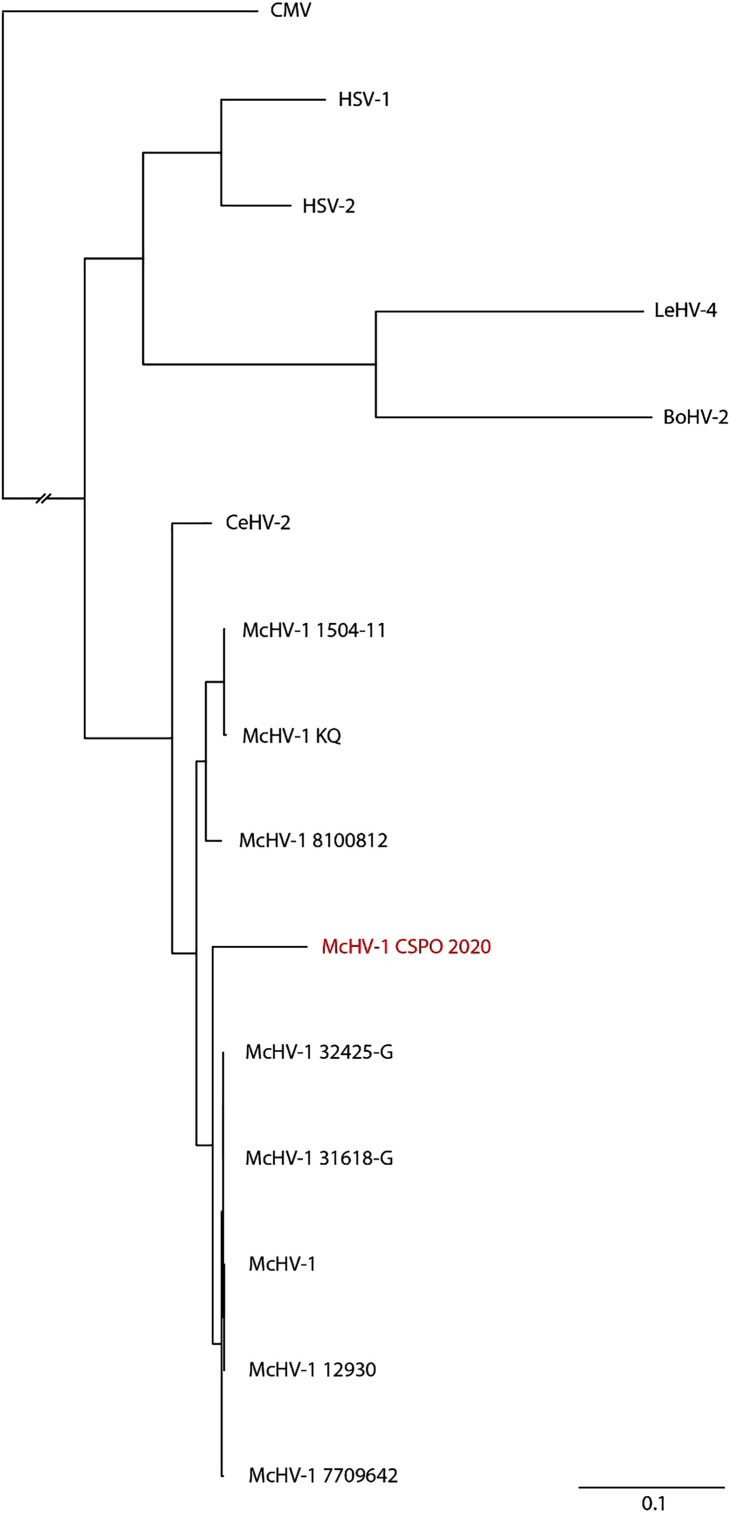
Phylogenetic relationship of herpes B virus. UL29 sequence from our isolate, the Clinique Saint-Pierre Ottignies 2020 isolate (McHV-1 CSPO 2020) was retrieved from the metagenomic sequencing data and aligned to selected herpes B virus (McHV-1) sequences, herpes simplex virus type 1 (HSV-1), herpes simplex virus type 2 (HSV-2), leporid alphaherpesvirus 4 (LeHV-4), cercopithecine alphaherpesvirus 2 (CeHV-2), bovine alphaherpesvirus 2 (BoHV-2), and cytomegalovirus (CMV) as an outgroup. Gaps were removed and a phylogenetic tree was constructed.

A comprehensive medical history, provided by the patient’s relatives, revealed an episode of lymphocytic meningitis 54 years prior, after a bite from a monkey that had been gifted to her. The monkey, purchased from a pet shop, died a few days after biting the patient. Medical information on this episode was ascertained and retrieved from the patient’s original medical files at the Saint-Pierre Hospital in Brussels, Belgium, where she was admitted in 1965 (Supplemental Figure 1). After receiving this information, CSF samples were tested for BV (McHV-1) DNA using a targeted PCR. *Macacine alphaherpesvirus* 1 genome was detected in CSF samples from days 3, 6, 10, and 24 (Table 1). Moreover, the presence of DNA was also confirmed in blood samples from days 4 and 6. Additional evidence of neuroinvasive infection was assessed via antibody index calculations comparing CSF and blood antibody fractions. Herpes simplex virus was used as proxy for McHV-1 due to their shared antigenic characteristics and VZV was used to make a comparison (Table 1).^17^ To confirm the McHV-1 finding, metagenomic sequencing was performed, which yielded 2.7 million nanopore reads. The consensus sequence showed McHV-1 isolate 7709642. Alignment of all the reads to this reference resulted in a coverage of 22.1% (Figure 2). From the DNA sequence of this isolate, the gene for the single-stranded DNA-binding protein (UL29) was aligned to HSV-1, HSV-2, leporid alphaherpesvirus 4 (LeHV-4), cercopithecine alphaherpesvirus 2 (CeHV-2), bovine alphaherpesvirus 2 (BoHV-2), McHV-1 sequences, and cytomegalovirus as an outgroup. After removing of gaps from this alignment, a phylogenetic tree was constructed (Figure 3). It showed that our isolate, the Clinique Saint-Pierre Ottignies (CSPO) isolate clustered with the representative genomes of McHV-1, but it was distinct from other clusters within the McHV-1 species.

**Table 1:** Herpes B virus diagnostics

Diagnostics	Day 3	Day 4	Day 6	Day 10	Day 24	Day 35	Day 105
CSF							
Herpes B virus (McHV-1) PCR (Ct value)	+ (29.3)		+ (28.6)	+ (32.1)	+ (42.4)	–	–
IgG anti-HSV (CSF/serum AI)					40.5	51.12	
IgG anti-VZV (CSF/serum AI)					21.88	32.17	
Blood/serum							
Herpes B virus PCR		+ (38.6)	+ (38.7)	–	–	–	

– = negative result; + = positive result; AI = IgG antibody index comparing antibodies in CSF and serum, corrected for albumin and total IgG levels. An AI > 3 is considered evidence of intrathecal antibody production; CSF = cerebrospinal fluid; Ct = cycle threshold; IgG anti-HSV = IgG antibodies directed against herpes simplex virus; IgG anti-VZV = IgG antibodies directed against varicella zoster virus; McHV-1 = *Macacine alphaherpesvirus* 1; PCR = polymerase chain reaction; VZV = varicella zoster virus.

After obtaining the PCR results, on day 14, acyclovir was switched to intravenous ganciclovir at a dosage of 5 mg/kg twice daily.^18^

On day 24, brain MRI showed a disappearance of the hyperintensities on DWI sequence. From day 29 onward, the patient’s neurological status improved. Body stiffness resolved, and the patient regained consciousness. She was extubated and transferred to the ward on day 31. PCR tests were conducted repeatedly, remaining positive in blood and CSF until days 6 and 24, respectively. No McHV-1 DNA was detected in the blood and CSF after days 10 and 35, respectively (Table 1). The patient exhibited significant clinical recovery and was able to speak and walk. She was transferred to a rehabilitation facility on day 48 and returned home on day 161. Her rehabilitation took place between days 48 and 208, with obvious improvement. On day 77, she displayed anosognosia, confusion, and verbal perseverations. On day 107, neuropsychological assessment identified a pronounced deficit in information processing speed and some impulsivity. Moreover, she was experiencing difficulties with working memory, interferences, and important deficits in the visual domain associated with prosopagnosia. Her executive functions were also altered. Mini-Mental State Examination was 23 out of 30 before and 30 out of 30 after rehabilitation. Starting from day 48, ganciclovir was switched to oral acyclovir 800 mg five times daily. We considered lifelong antiviral prophylaxis with acyclovir.^18^

## MATERIALS AND METHODS

### BV detection.

Clinical samples were spiked with phocine herpesvirus 1 to serve as an internal control for DNA virus detection. Total nucleic acids (NAs) were extracted directly from 200 µL of clinical material, using the MagNAPure 96 DNA and Viral NA Small Volume Kit (Roche Diagnostics, Almere, the Netherlands) with 100 µL output eluate.^19^ Extracted NAs were tested by a real-time PCR assay designed to detect known McHV-1.^20^

### Metagenomic sequencing.

DNA was isolated using an in-house method.^21^ In short, the sample was added to a lysis buffer, after which the mixture was incubated with magnetic beads. Beads bound to DNA were extracted using a magnetic block and eluted using PCR-grade water. A metagenomics library was generated using the PCR Sequencing Kit from Oxford Nanopore Technologies (Oxford, United Kingdom; SQK-PSK004) and sequenced on a R9.4 flow cell.

### Sequence data analysis.

The resulting sequence data were aligned to the reference McHV-1 (accession no. NC_004812) using the CLC Genomics Workbench 21 (Qiagen, Hilden, Germany). From this alignment, a consensus sequence was extracted and used in a BLASTn query. All reads were realigned to the best BLASTn score McHV-1 isolate 7709642 (accession no. KY628982.1), and a concatenated consensus sequence was extracted from this alignment using regions with > 3 read coverage. The UL29 sequence was submitted to GenBank (accession no. OL513439).

### Phylogenetic analysis.

The extracted CPSO UL29 nucleotide sequence was aligned to McHV-1 isolates E2490 (KY628984), McHV-1 RefSeq (NC_004812), McHV-1 isolate 32425-G (KY628981), McHV-1 isolate 12930 (KY628971), McHV-1 isolate 31618-G (KY628978), McHV-1 isolate KQ (KY628970), McHV-1 isolate 1504-11 (KY628969), McHV-1 isolate 8100812 (KY628968), McHV-1 isolate 7709642 (KY628982), HSV-1 RefSeq (NC_001806), HSV-2 RefSeq (NC_001798), CeHV-2 RefSeq (NC_006560), LeHV-4 RefSeq (NC_029311), BoHV-2 strain Riems 8/85 (MT862164), and human betaherpesvirus 5 (HHV-5 RefSeq (NC_006273) using the ClustalW software running within the BioEdit (version 7.0.5.3) program.^22^ The alignment was manually checked for discrepancies, after which IQ-TREE was used to perform a maximum-likelihood phylogenetic analysis under the phylogenetic tree model (TVM + F + G4) as the best predicted model using the ultrafast bootstrap option with 1,000 replicates.^23^

## DISCUSSION

Here we report for the first time that BV can reactivate in humans after 54 years of latency, causing severe meningoencephalitis. BV is commonly found in macaque monkeys, with a prevalence ranging from 70% to nearly 100% in both wild and captive adult macaques.^13^ The macaque host is found most often in the Asian wilds, but colonies of these animals have been exported.^13^ BV may be considered as the macaque equivalent of HSV, having coevolved with its natural macaque host.^13^ Perpetuation of the virus within the nervous system has minimal adverse effects on the natural host, although fatal infection has been shown among infant captive macaques.^13^ However, occasional shedding of the infectious virus can occur, allowing transmission to naive hosts such as other monkey species housed next to macaques^1^ or humans.^13^ In these cases, the neurovirulence of BV becomes evident.^13^^,^^24^ Most exposures have been associated with bites or scratches from captive laboratory-housed macaques.^13^ Since the first report describing a human infection by BV in 1933,^15^^,^^25^ the virus has been linked to more than 20 reported human deaths and a limited number of survivors.^1^^,^^2^^,^^5^^–^^12^ Exact infection rates are unknown, and reports of latency in humans are scarce.

Reactivation of BV, in both wild and captive macaques,^3^ occurs in response to various stressful stimuli, such as social/housing challenges, transportation, immunosuppression and seasonal breeding.^13^ Although reactivation in humans has been suggested, reports have not yet been supported by laboratory analysis. Some cases following trivial exposure have been described, and in other instances, multiple exposures occurred throughout time, although infected persons could not recall recent exposures.^15^^,^^26^ As an example, in 1973, a patient presenting with signs of ophthalmic zoster was diagnosed with BV through virus isolation and serology, but the patient’s last documented exposure to primates had taken place more than 10 years before symptom development.^9^ In 2011, another case presented ocular manifestations with vitreous samples positive for BV DNA after a previous episode of BV meningitis and encephalitis in 1981.^27^ Finally, a recent publication noted that several more cases have not been published but were documented clinically and with laboratory evaluations using serology and molecular testing.^28^ Nevertheless, a laboratory-confirmed reactivation with neuroinvasive disease after a latent period of as long as 54 years has not been described until now.

In this case, the patient presented with meningitis at 11 years of age after a bite from a gifted monkey. She recovered completely and had no subsequent exposure to monkeys in her life. She was admitted to the hospital at age 66 years with viral meningoencephalitis, and BV DNA was detected in her CSF samples and blood samples.

Only a limited number of BV sequences are known. Phylogenetic analysis of the virus revealed a clearly distinct BV compared with that of other known BVs. To determine the route of evolution of this BV isolate, a viral sequence from the initial infection is crucial. Unfortunately, material has not been stored, and sequences from circulating BVs during this period are not available.

Altogether, this case illustrates how a latent BV infection in a human can reactivate and cause a neuroinvasive disease after 54 years and without evidence of immunodepression. Thus, clinicians should be aware that BV can reactivate in humans, leading to severe neurological disease, and should consider BV as part of the differential diagnoses when a patient’s medical history reveals exposure to monkeys, even if the exposure occurred many years earlier. The optimal duration of antiviral prophylaxis after BV central nervous system infection remains controversial.^18^ However, this case provides support for lifelong antiviral prophylaxis in these patients.

## Supplemental files

10.4269/ajtmh.23-0253 Supplemental Materials
