# Supplementary material for: Case Report: Late Reactivation of Herpes B Virus After a Monkey Bite: A Case of Severe Meningoencephalitis
Source: Am J Trop Med Hyg. 2023 Oct 16;109(6):1277–81. doi: 10.4269/ajtmh.23-0253 (PMC10793070; doi:10.4269/ajtmh.23-0253)
Supplement: Supplemental Materials [file tpmd230253.SD1.pdf]

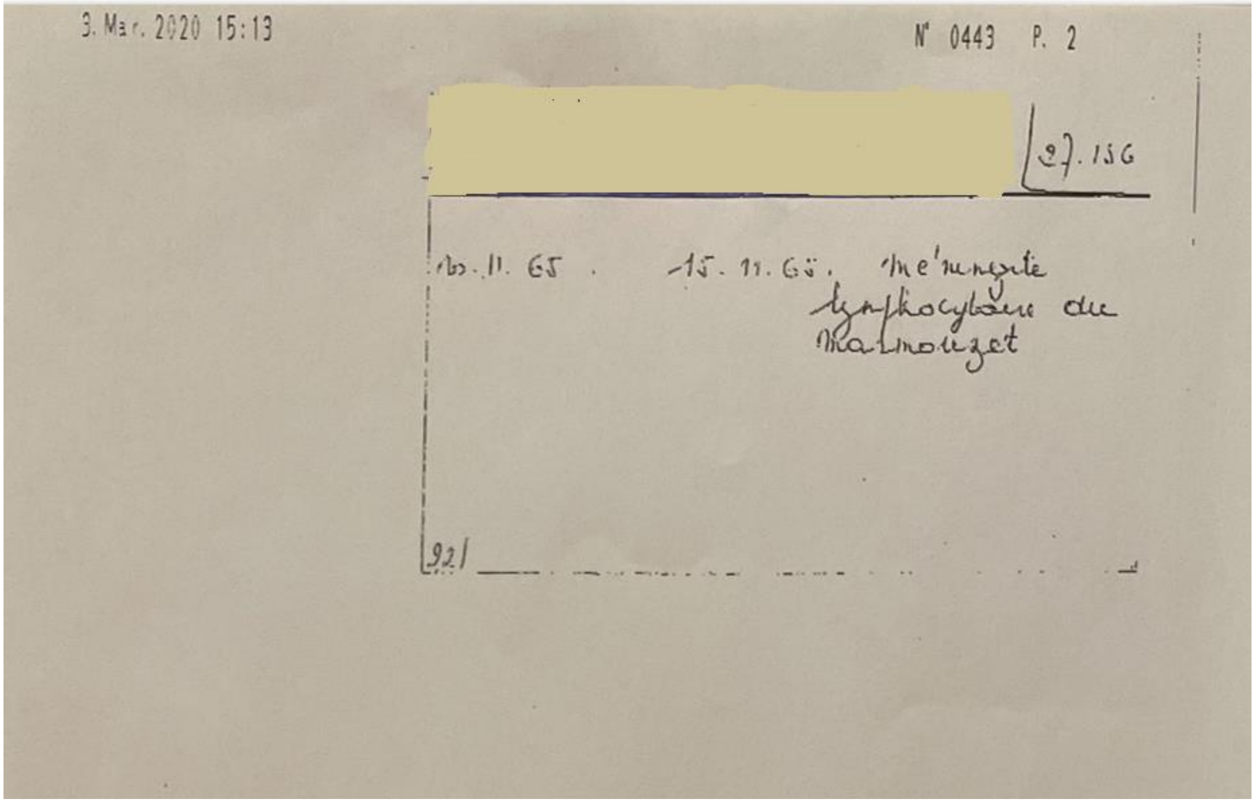
3. Mar. 2020 15:13

N° 0443 P. 2

[27].156

10.11.65. 15.11.65. méningite lymphocytaire de Marmouzet

921

A legend could be added, as it is added in the original manuscript:
Supplementary figure 1. Patient's original medical file at the Saint-Pierre Hospital in Brussels, Belgium

Supplementary Table S1. All diagnostics performed on the patients' samples.

	Site	Analysis	Day 3	Day 4	Day 6	Day 10	Day 24	Day 35	Day 105
**Appearance**	CSF		Opalescent		Clear	Clear	Clear	Clear	
**RBCs (No/mm^3)**	CSF		18		2	14	13	0	2
**WBCs (No/mm^3)**	CSF		128		174	388	27	31	4
**Neutrophils %**	CSF		3		0	0	0	0	
**Lymphocytes %**	CSF		78		83	97	89	98	
**Mono-macrophage %**	CSF		19		17	3	11	2	
**Eosinophils %**	CSF		0		0	0	0	0	
**Basophils %**	CSF		0		0	0	0	59	
**Proteins (mg/dL)**	CSF		74		144	112	87	59	35
**Glucose (mg/dL)**	CSF		63		42	50	62	58	54
**Culture**	CSF		STER		STER	STER	STER	STER	
Escherichia coli	CSF	PCR			NEG				
Haemophilus influenzae	CSF	PCR			NEG				
Listeria monocytogenes	CSF	PCR			NEG				
Neisseria meningitidis	CSF	PCR			NEG				
Streptococcus agalactiae	CSF	PCR			NEG				
Streptococcus pneumoniae	CSF	PCR			NEG				
Borrelia burgdorferi	CSF	ST	NEG						
Cytomegalovirus	CSF	PCR			NEG				
Enterovirus	CSF	PCR			NEG				
Herpes simplex virus 1	CSF	PCR	NEG		NEG	NEG			
Herpes simplex virus 2	CSF	PCR	NEG		NEG	NEG			
Human herpesvirus 6	CSF	PCR			NEG				
Human parechovirus	CSF	PCR			NEG				
	**Site**	**Analysis**	**Day 3**	**Day 4**	**Day 6**	**Day 10**	**Day 24**	**Day 35**	**Day 105**
Varicella zoster virus	CSF	PCR	NEG		NEG	NEG			

***Puumala Orthohantavirus* IgG**	CSF	ST	NEG						
***Puumala Orthohantavirus* IgM**	CSF	ST	NEG						
**Herpes B virus**	**CSF**	PCR	**POS**		**POS**	**POS**	**POS**	NEG	NEG
**Herpes B virus**	**Blood**	PCR		**POS**	**POS**	**NEG**	NEG	NEG	
**Leptospiral strain**	**CSF**	**ST**							
Grippotyphosa			NEG						
Javanica			NEG						
Canicola canicola			NEG						
Hebdomadis			NEG						
Ballum			NEG						
Pomona proechimys			NEG						
Icterohaemorrhagiae			NEG						
Sejroe Hardjo			NEG						
Semaranga patoc			NEG						
RT-QuIC						NEG			
Mycobacterium tuberculosis						NEG			

RBC: red blood cells.

WBC: white blood cells.

PCR: polymerase chain reaction.

ST: serological test.

STER: sterile culture.

RT-QuIC: real-time quaking-induced conversion (for diagnosis of Creutzfeldt–Jakob disease).

NEG: negative result.

POS: positive result.

Puumala Orthohantavirus **IgG:** antibodies IgG anti Puumala Orthohantavirus.

Puumala Orthohantavirus **IgM:** antibodies IgM anti Puumala Orthohantavirus.
